# Spirodalesol analog 8A inhibits NLRP3 inflammasome activation and attenuates inflammatory disease by directly targeting adaptor protein ASC

**DOI:** 10.1016/j.jbc.2022.102696

**Published:** 2022-11-12

**Authors:** Wen Liu, Jiashu Yang, Shihao Fang, Chenyang Jiao, Jianhua Gao, Aihua Zhang, Tiancong Wu, Renxiang Tan, Qiang Xu, Wenjie Guo

**Affiliations:** 1 State Key Laboratory of Pharmaceutical Biotechnology, School of Life Sciences, Nanjing University, Nanjing, China; 2 Chemistry and Biomedicine Innovation Center, Nanjing University, Nanjing, China; 3 Jiangsu Province Engineering Research Center for Marine Bio-resources Sustainable Utilization, Hohai University, Nanjing, China; 4 Department of Radiation Oncology, Jinling Hospital, Nanjing University, School Medicine, Nanjing, China

**Keywords:** spirodalesol analog, NLRP3 inflammasome, ASC, sepsis, gouty arthritis, ASC, Apoptosis-associated speck-like protein containing a CARD, BMDM, bone marrow-derived macrophage, CETSA, Cellular thermal shift assay, IF, immunofluorescence, IHC, Immunohistochemistry, LPS, lipopolysaccharide, MST, Microscale thermophoresis assay, MSU, monosodium urate, NLR, NOD-like receptor, NLRP3, Nod-like receptor family protein 3, PYD, pyrin domain, PMA, phorbol myristate acetate, ROS, reactive oxgen species

## Abstract

Pharmacological inhibition of the Nod-like receptor family protein 3 (NLRP3) inflammasome contributes to the treatment of numerous inflammation-related diseases, making it a desirable drug target. Spirodalesol, derived from the ascomycete fungus *Daldinia eschscholzii*, has been reported to inhibit NLRP3 inflammasome activation. Based on the structure of spirodalesol, we synthesized and screened a series of analogs to find a more potent inhibitor. Analog compound 8A was identified as the most potent selective inhibitor for NLRP3 inflammasome assembly, but 8A did not inhibit the priming phase of the inflammasome. Specifically, while 8A did not reduce NLRP3 oligomerization, we found that it inhibited the oligomerization of adaptor protein apoptosis-associated speck-like protein containing a caspase activation and recruitment domain (ASC), as ASC speck formation was significantly reduced. Also, 8A interrupted the assembly of the NLRP3 inflammasome complex and inhibited the activation of caspase-1. Subsequently, we used a cellular thermal shift assay and microscale thermophoresis assay to demonstrate that 8A interacts directly with ASC, both *in vitro* and *ex vivo*. Further, 8A alleviated lipopolysaccharide-induced endotoxemia, as well as monosodium urate-induced peritonitis and gouty arthritis in mice by suppressing NLRP3 inflammasome activation. Thus, 8A was identified as a promising ASC inhibitor to treat inflammasome-driven diseases.

Inflammasomes are multiprotein signaling complexes that control inflammatory responses by regulating the proteolytic enzyme caspase-1 (1). Inflammasomes are assembled in the cytosol of host cells to detect exogenous pathogenic or endogenous warning signals that, in turn, activate inflammatory caspases that promote cytokine production and induce pyroptotic cell death (2). Based on the structural features and unique activators, the inflammasomes are divided and named NOD-like receptor (NLR), AIM2-like receptor family members, and pyrin (3). The canonical inflammasome complex consists of a cytosolic sensor (NLR or AIM2-like receptor protein), adaptor protein ASC [apoptosis-associated speck-like protein containing a caspase activation and recruitment domain], and effector caspase-1 (4). As the central adapter that bridges the upstream sensors to procaspase-1, ASC is composed of pyrin domain (PYD) and caspase activation and recruitment domain, and both domains promote homotypic interactions (5). Procaspase-1 is subsequently activated, leading to the cleavage of pro-IL-1β, pro-IL-18, and gasdermin-D in their mature forms, which drives pyroptosis (6, 7).

Nod-like receptor family protein 3 (NLRP3), a member of the NLR family, consists of an N-terminal PYD domain, a central NACHT domain that mediates ATP hydrolysis, and a C-terminal leucine-rich repeat domain. As an innate immune sensor, NLRP3 responds to many stimuli derived from exogenous pathogens or endogenous danger signals (8). A recent study showed that inactive NLRP3 forms a double ring of 12 to 16 monomers that shield its pyrin domains from the cytosol. Before activation, NLRP3 interacts with the protein NEK7, which facilitates NLRP3 signaling. Upon activation, NLRP3 assembles an inflammasome complex *via* PYD-PYD interactions with ASC, forming a platform for caspase-1 dimerization and activation (9, 10). On the one hand, its activation may protect against infectious pathogens. On the other hand, over- or long-lasting activation of the NLRP3 inflammasome has been implicated in the development of a range of human acute or chronic inflammatory disorders, including inflammatory bowel disease, gout, atherosclerosis, diabetes, neurodegenerative disease, depression, and cancer (11, 12, 13, 14). Accordingly, NLRP3 is considered a highly desirable therapeutic target for these diseases (15).

In this study, we identified a novel ASC inhibitor, 8A, that binds to and attenuates ASC in the nucleus and prevents its recruitment by NLRP3, thereby reducing caspase-1 activation and the maturation of IL-1β. This working model may improve the treatment of NLPR3-related diseases, including sepsis and gouty arthritis.

## Results

### 8A reduces IL-1β secretion in macrophages

Previously, spirodalesol derived from *Daldinia eschscholzii* was shown to inhibit NLRP3 inflammasome activation and improve sepsis (16). Based on these observations, we synthesized and screened a series of spirodalesol derivates to identify more potent compounds with simple structures. The structures of these compounds and their inhibitory activities against IL-1β are shown in Fig. S1, *A–C*.

**Figure 1:**
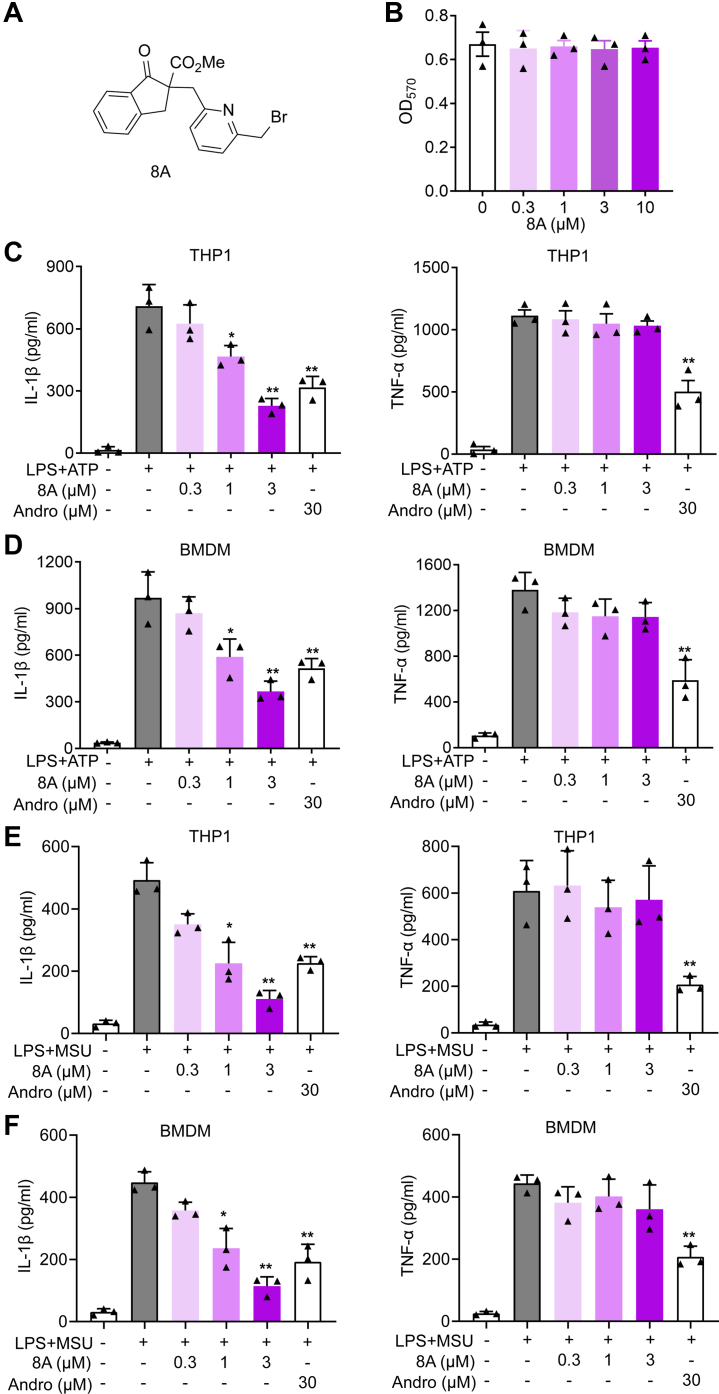
**8A selectively inhibited IL-1β secretion.***A*, chemical structure of 8A. *B*, PMA-differentiated THP1 cells were treated with 8A with the indicated concentrations. After 24 h, cell viability was determined by MTT assay. *C*, PMA-differentiated THP1 cells and (*D*) BMDMs were stimulated with 100 ng/ml LPS for 3 h, followed by indicated concentrations of 8A treatment for 1 h and then another 1 h of 5 mM ATP stimulation. IL-1β and TNF-α in supernatants were determined by ELISA. *E*, PMA-differentiated THP1 cells and (*F*) BMDM were stimulated with 100 ng/ml LPS for 3 h, followed by indicated dose of 8A treatment with or without 500 μg/ml MSU for 2 h. IL-1β and TNF-α in supernatant were determined by ELISA. Andro: andrographolide. Data were presented as mean ± SD of three independent experiments. ∗*p* < 0.05, ∗∗*p* < 0.01 *versus* LPS + ATP. BMDMs, bone marrow-derived macrophages; LPS, lipopolysaccharide; MSU, monosodium urate; PMA, phorbol myristate acetate.

Among these derivatives, compounds 7 and 8A showed almost the same potency. The most remarkable difference was that compound 8A inhibited IL-1β more efficiently than compound 7 at a concentration of 1 μM (Fig. S1, *A–C*). However, in our study, we found that the solubilities of these compounds were different. Precipitated solid was observed when the buffer solution of compound 7 was prepared at a concentration of 100 μM in the culture medium but was not found with compound 8A. Therefore, we selected compound 8A for further studies. As shown in Figure 1, Figure 8, *B*–*D*, 8A suppressed IL-1β secretion in bone marrow-derived macrophages (BMDMs) and THP1-derived macrophages triggered by lipopolysaccharide (LPS) + ATP without affecting cell viability or TNF-α secretion. Its potency was superior to the previously reported activity of andrographolide, an NLRP3 inflammasome inhibitor (17). Moreover, LPS + monosodium urate (MSU)–induced IL-1β secretion, although not that of TNF-α, was inhibited by 8A (Fig. 1, *E* and *F*).

### 8A inhibits caspase-1 activation in macrophages

**Figure 2:**
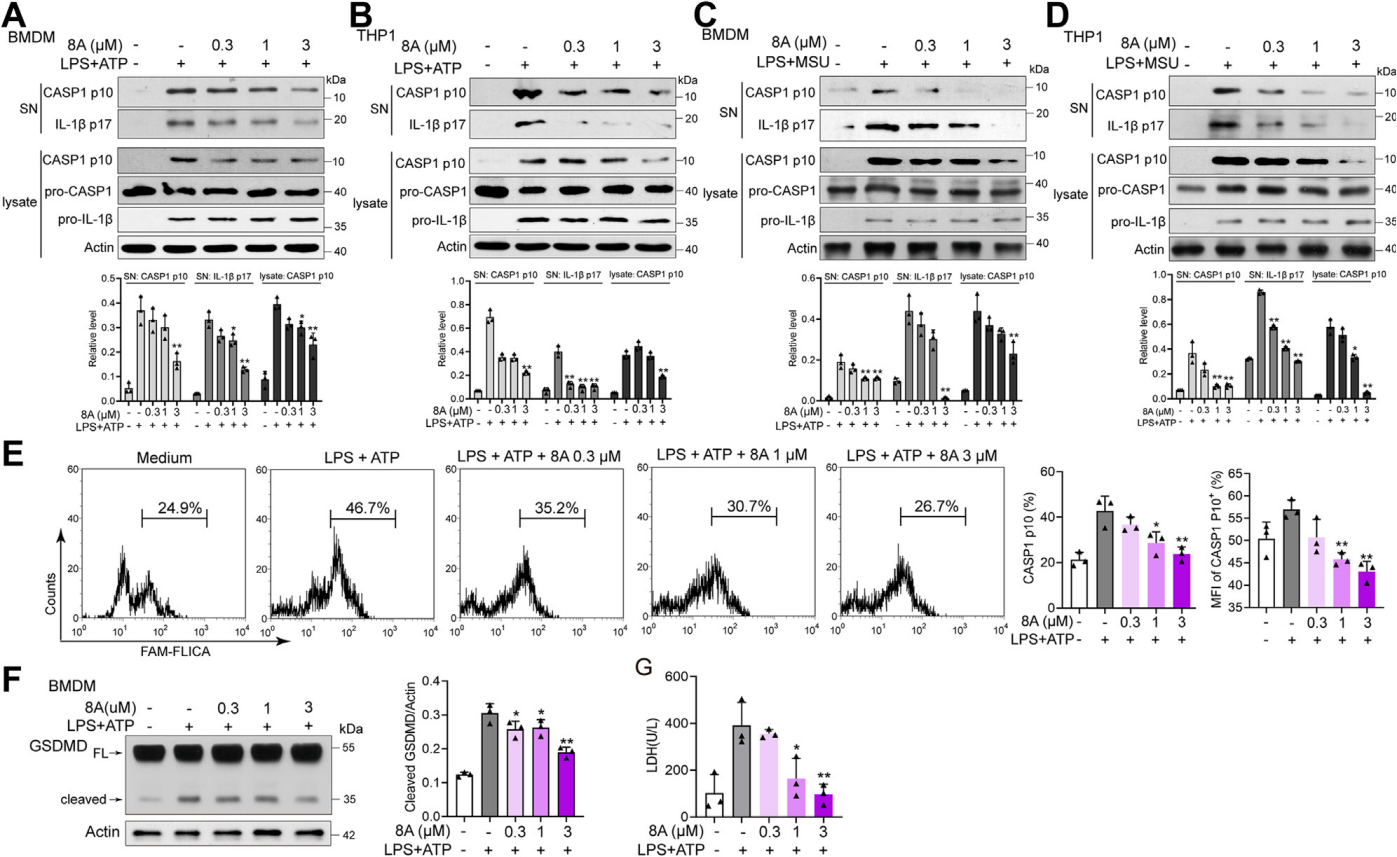
**8A inhibited CASP1 activation.***A* and *B*, LPS-primed THP1 cells or BMDM cells were treated with the indicated concentrations of 8A for 1 h, followed by ATP (5 mM) stimulation for 1 h. Cell lysates were collected and subjected to western blotting. *C* and *D*, LPS-primed THP1 cells or BMDM cells were treated with indicated concentrations of 8A for 1 h, followed by MSU (500 μg/ml) stimulation for 2 h. Cell lysates were collected and subjected to Western blotting. *E*, CASP1 activation was determined by FAM-FLICA staining. *F* and *G*, LPS-primed BMDM cells were treated with the indicated concentrations of 8A for 1 h, followed by ATP (5 mM) stimulation for 1 h. The expression of GSDMD in cell lysates and LDH in cell culture was examined. Data were representative of three independent experiments. Results were presented as the mean ± SD of three independent experiments. ∗*p* < 0.05, ∗∗*p* < 0.01 *versus* LPS + ATP. BMDMs, bone marrow-derived macrophages; GSDMD, gasdermin-D; LDH, lactate dehydrogenase; LPS, lipopolysaccharide; MSU, monosodium urate.

We subsequently sought to elucidate the role of 8A against inflammasome activation. We observed that LPS + ATP- or MSU-triggered elevation of caspase-1 p10 and IL-1β p17 levels in culture supernatants were significantly suppressed by 8A (Fig. 2, *A*–*D*). Moreover, the pro-IL-1β levels remained unaltered (Fig. 2, *A*–*D*). Activation of CASP1 was confirmed by FAM-FLICA staining (Fig. 2*E*). We also established that the cleavage of gasdermin-D and release of lactate dehydrogenase, downstream of caspase-1 activation induced by LPS + ATP, were downregulated by 8A (Fig. 2, *F* and *G*). These findings indicate the high potency of the anti-inflammatory action exhibited by 8A and that 8A might selectively inhibit the assembly phase, although not the priming phase, of NLRP3 inflammasome activation. To confirm this conjecture, we examined the phosphorylation of NF-κB and LPS-induced expression of NLRP3. As shown, 8A had no appreciable effect on LPS-induced phosphorylation of NF-κB or NLRP3 expression (Fig. S2).

### 8A interrupts the assembly of the NLRP3/ASC/caspase-1 complex

**Figure 3:**
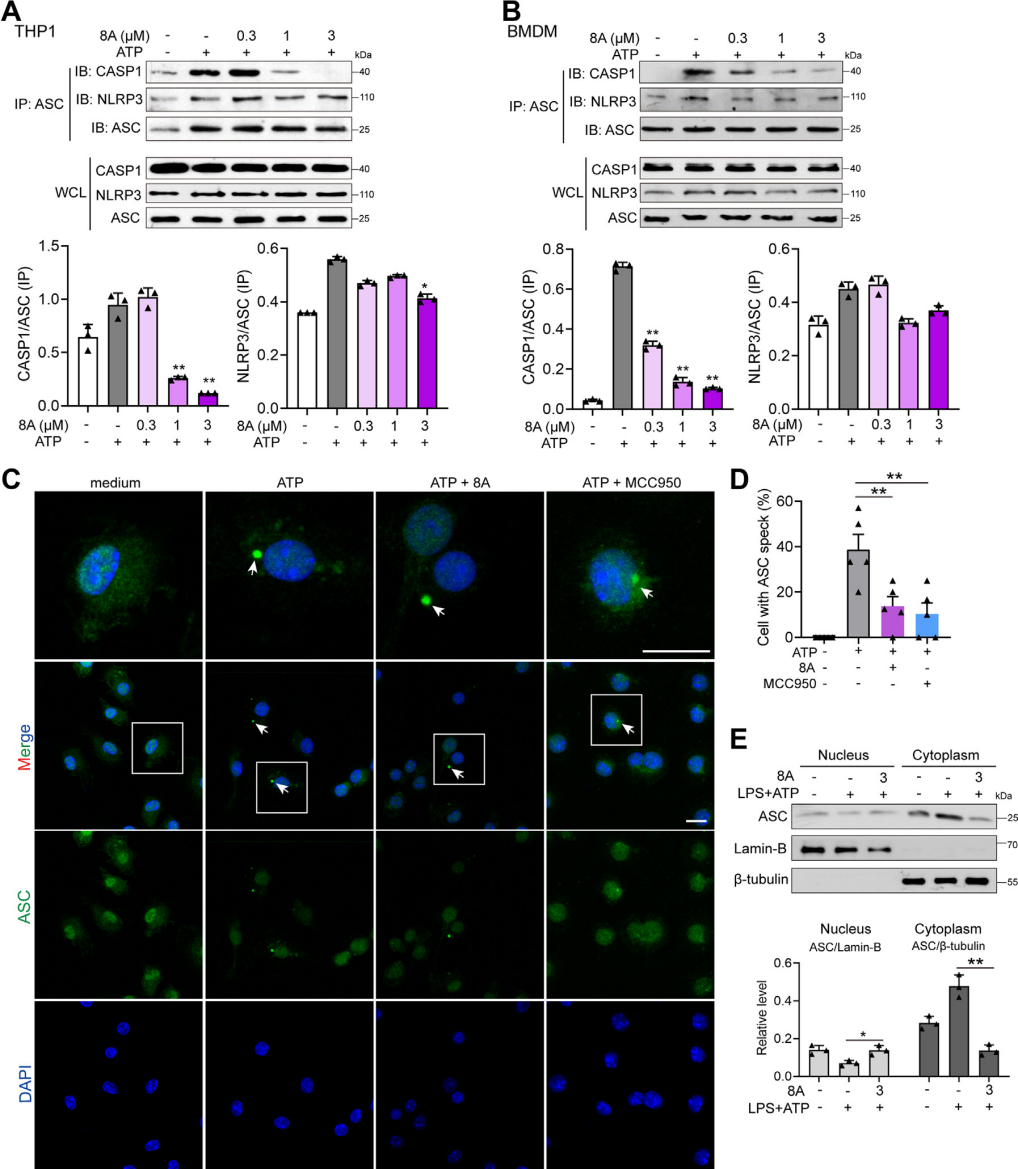
**8A inhibited NLRP3/ASC/pro-CASP1 assembly.***A* and *B*, LPS-primed THP1 cells or BMDM cells were treated with the indicated concentrations of 8A for 1 h, followed by ATP stimulation for 15 min. Cell lysates were collected for co-immunoprecipitation (co-IP) assay. *C* and *D*, LPS-primed BMDMs were treated with 8A (3 μM) or MCC950 (0.3 μM) for 1 h, followed by ATP stimulation for 15 min. ASC speck formation was determined using immunofluorescence. Cells with ASC speck (indicated by *white arrow*) were counted in five fields from every group and expressed as the mean ± SD. Scale bar 10 μm. *E*, LPS-primed BMDM cells were treated with 8A (3 μM) for 1 h, followed by ATP stimulation for 30 min. The nuclear and cytosolic components were separated and subjected to Western blotting. Data in *A–C* and *E* were representative of three independent experiments. Results were presented as mean ± SD of three independent experiments. ∗*p* < 0.05, ∗∗*p* < 0.01 *versus* the ATP group or as indicated. ASC, apoptosis-associated speck-like protein containing a caspase activation and recruitment domain; BMDM, bone marrow-derived macrophage; LPS, lipopolysaccharide; MSU, monosodium urate; NLRP3, Nod-like receptor family protein 3.

We examined the process of NLRP3 inflammasome formation using immunoprecipitation and immunofluorescence (IF). We observed that ATP stimulation-induced formation of the NLRP3/ASC/caspase-1 complex was interrupted by 8A in a dose-dependent manner (Fig. 3, *A* and *B*). Moreover, IF analysis revealed that ATP induced formation of ASC specks (indicated by white arrow), whereas 8A and MCC950 treatment reduced the number of specks and contributed to the retention of ASC within the nuclei (Fig. 3, *C* and *D*). This phenomenon was further confirmed by examining ASC levels in the nuclear and cytosolic fractions. As shown in Figure 3, Figure 8*E*, 8A treatment resulted in the retention of ASC in the nucleus. We transfected ASC-GFP plasmid into 293T cells with or with NLRP3 plasmid to further examine speck formation as well as the localization of ASC. As shown in Fig. S3, *A* and *B*, GFP diffused throughout the whole cell in the GFP vector-transfection group. We estimated that approximately 20% of cells spontaneously formed ASC specks without NLRP3-encoding plasmid transfection and that about 70% of cells produced ASC specks when co-transfected with NLRP3-encoding plasmid (indicated by white arrow). In addition, ASC was partially localized in the nuclei without NLRP3 induction (indicated by red arrow). Both 8A and MCC950 treatment reduced the formation of ASC speck in 293T cells and restrained part of the ASC in the nuclei (red arrow). These results further confirmed that 8A reduced ASC speck formation and inhibited the activation of caspase-1.

### 8A specifically targets ASC

**Figure 4:**
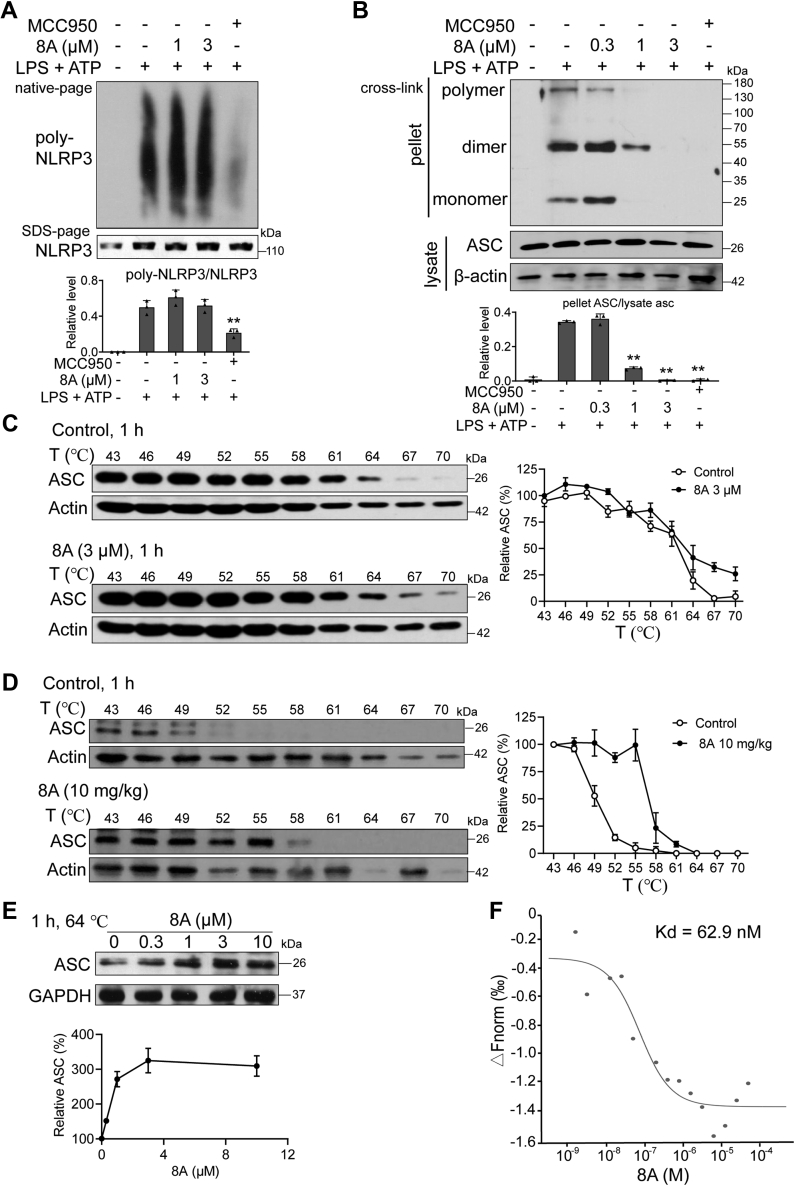
**8A inhibited ASC oligomerization *via* direct interaction.***A* and *B*, LPS-primed BMDM were treated with 8A for 1 h, followed by ATP stimulation for 15 min. *A*, oligomerization of NLRP3 was analyzed using *blue* native PAGE. *B*, oligomerization of ASC in whole lysates was examined using Western blotting. *C*, BMDMs were incubated with 8A (DMSO as control) for 1 h, and cells were collected and subjected for CETSA. *D*, mice were i.g. with 10 mg/kg 8A once a day for 3 days. Peritoneal macrophages were collected and subjected to CETSA. *E*, BMDM cells were incubated with indicated concentrations of 8A for 1 h, and cells were collected subjected for CETSA at 64 °C. *F*, lysates from 293T cells transfected with ASC-GFP plasmid were used to determine the binding between ASC and 8A. Data were representative of three independent experiments. Results were presented as the mean ± SD of three independent experiments. ∗*p* < 0.05, ∗∗*p* < 0.01 *versus* ATP group or as indicated. ASC, apoptosis-associated speck-like protein containing a caspase activation and recruitment domain; BMDM, bone marrow-derived macrophage; CETSA, cellular thermal shift assay; LPS, lipopolysaccharide; NLRP3, Nod-like receptor family protein 3.

It is well established that mitochondrial dysfunction, exemplified by the overproduction of reactive oxgen species (ROS), is essential for the assembly of the NLRP3 inflammasome (18, 19). As detected by DCFH-DA staining, we found that ATP treatment induced high-level ROS generation. ROS production was slightly affected by 8A (Fig. S4, *A* and *B*), thereby indicating that the inhibitory activity of 8A is mediated downstream of mitochondrial and ROS signaling. Neither 8A or MCC950 altered the level of NLRP3 in SDS-PAGE (Fig. 4*A*). However, oligomerization of NLRP3 was significantly inhibited by MCC950 but not 8A. Moreover, ASC oligomerization was significantly reduced by treatment with 8A (Fig. 4*B*). These results indicate that ASC recruitment by NLRP3 is interrupted by 8A.

Based on our observation that 8A reduced the cytosolic translocation and oligomerization of ASC, we examined the binding between 8A and ASC. We employed the cellular thermal shift assay (CETSA), a recently described method that facilitates the rapid and simple assessment of the target engagement of drugs in a cellular context. In vehicle-treated cells, the degradation of ASC commenced at 64 °C and was complete at 67 °C, whereas in 8A-treated cells, ASC was still detectable at 70 °C (Fig. 4*C*). Furthermore, we found that 8A dose-dependently enhanced the levels of ASC at 64 °C, indicating an enhanced stability of ASC in response to 8A treatment (Fig. 4*C*). We also subjected peritoneal macrophages isolated from 8A-treated mice to CETSA to confirm that 8A can also bind to ASC after administration in mice. Accordingly, we detected an interaction between 8A and ASC (Fig. 4*E*). Furthermore, the microscale thermophoresis assay (MST) was used to quantify the interaction between ASC and 8A. MST showed that 8A interacted with the ASC with a dissociation constant (Kd) of 62.9 nM (Fig. 4*F*).

As ASC is also involved in activating the AIM2 inflammasome, we wondered whether 8A affects AIM2 inflammasome-mediated IL-1β release. Therefore, we examined the effect of 8A on LPS + poly(dA:dT)-induced IL-1β release. Both 8A and MCC950-induced IL-1β induced by LPS + ATP, while only 8A inhibited LPS + poly(dA:dT)-induced IL-1β as a known AIM2 inhibitor shikonin did (Fig. S5, *A* and *B*) (20). Combined with the data on the 8A-ASC interaction, we believe that 8A is an ASC inhibitor.

### 8A alleviates LPS-induced endotoxemia in mice by inhibiting NLRP3 inflammasome activation

**Figure 5:**
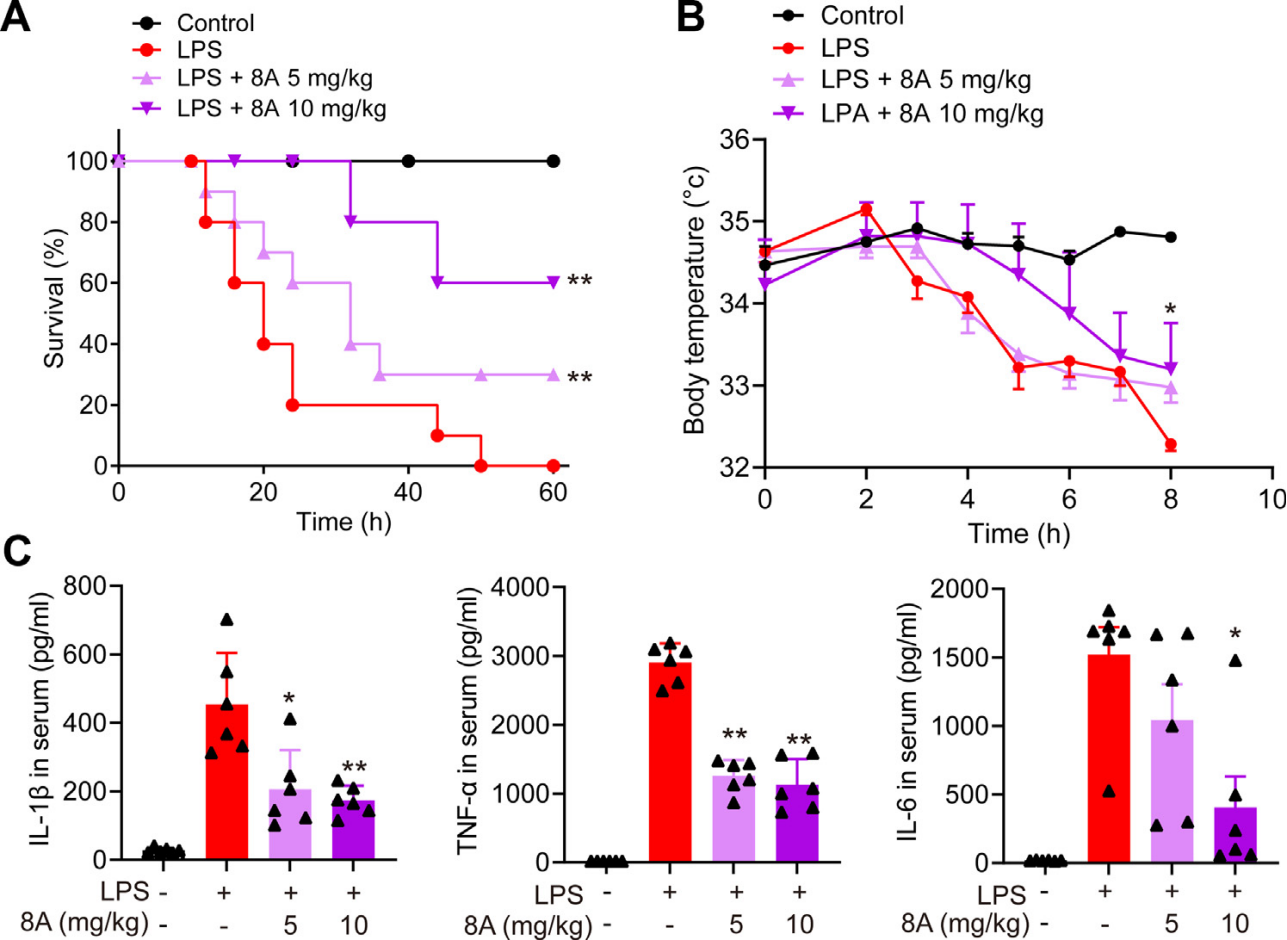
**8A ameliorated LPS-induced endotoxemia in mice.***A*, mice were treated with the indicated doses of 8A (i.g.) once after the LPS challenge (10 mg/kg, i.p.). *B*, survival rates and body temperature were recorded. *C*, after LPS challenge with or without 8A treatment for 24 h, mice were sacrificed. Levels of cytokines in serum were measured. There were 10 mice in *A* and *B*. Data in *C* were presented as the mean ± SD of 6 mice. ∗*p* < 0.05, ∗∗*p* < 0.01 *versus* LPS alone treated group. LPS, lipopolysaccharide.

**Figure 6:**
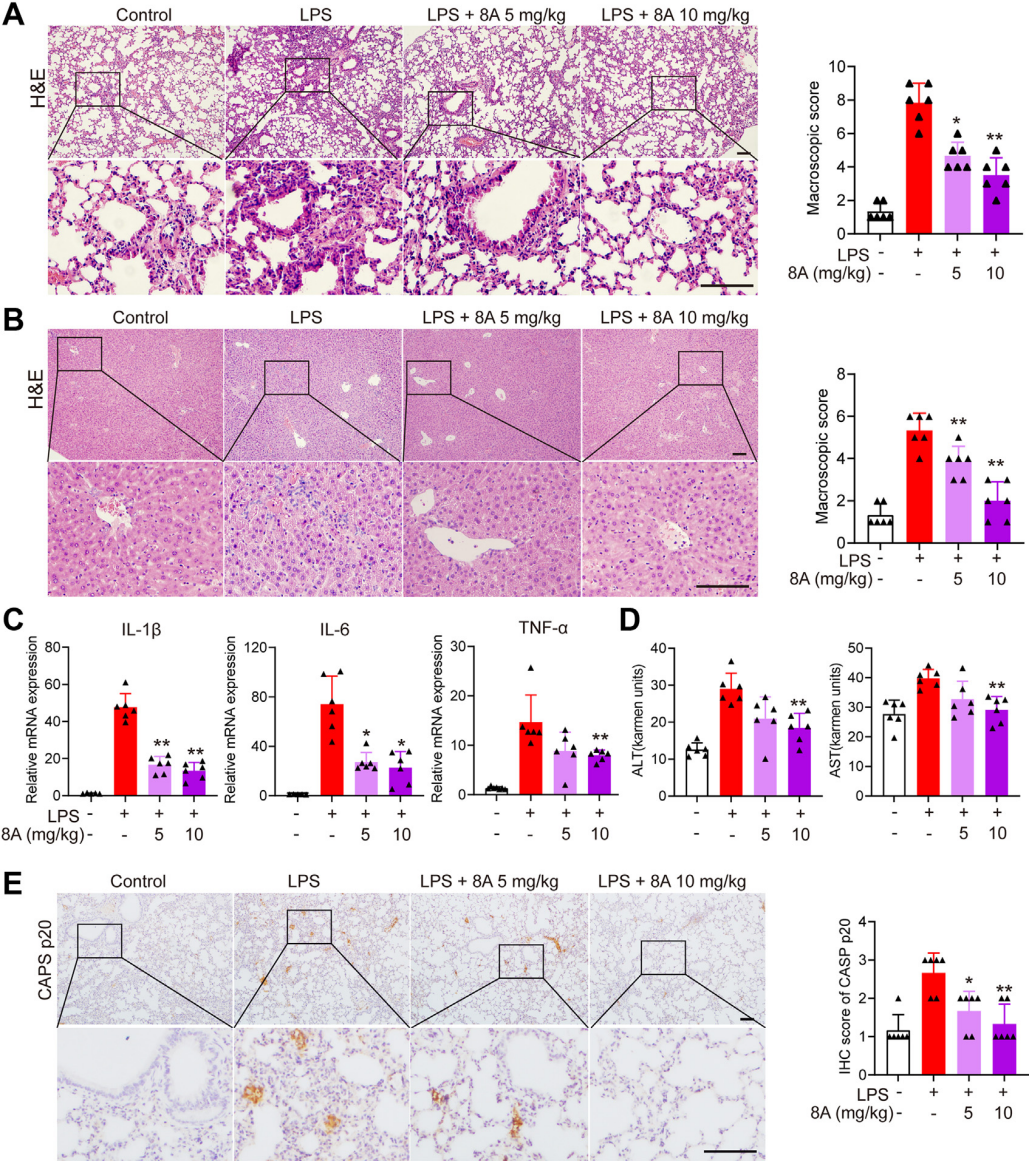
**8A decreased lung and liver damage in mice with LPS-induced endotoxemia.** After the LPS challenge for 24 h, mice were sacrificed. *A*, representative mouse lung tissue sections were stained with H&E; *B*, mouse liver tissue sections were stained with H&E. *C*, levels of pro-inflammatory cytokines in the lung tissue were determined by RT-PCR. *D*, ALT and AST levels. *E*, Representative mouse lung tissue sections were subjected to IHC staining for CASP p20. Scale bar 50 μm in *A*, *B* and *E*. Data in *C* and *D* were presented as the mean ± SD of six mice. Data in *A*, *B* and *E* were expressed as the mean ± SD of five fields per mouse in each group. ∗*p* < 0.05, ∗∗*p* < 0.01 *versus* LPS alone treated group. ALT, alanine transaminase; AST, aspartate aminotransferase; IHC, immunohistochemistry; LPS, lipopolysaccharide.

To confirm the therapeutic effect of 8A on NLRP3 inflammasome-related diseases, we used an LPS-induced murine endotoxemia model and found that 8A significantly increased the survival of mice with sepsis (Fig. 5*A*). The LPS-induced reduction in shell body temperature was also reversed by 8A treatment (Fig. 5*B*). Overproduction of inflammatory cytokines is believed to be an important factor contributing to the development of severe sepsis. We found that the administration of 8A inhibited the secretion of proinflammatory cytokines such as IL-1β and IL-6 in the serum (Fig. 5*C*). Moreover, we established that both lung and liver injuries caused by LPS were markedly alleviated in 8A-treated mice (Fig.6, *A* and *B*), indicated by reduced inflammatory cell infiltration in the liver (Fig. 6*C*) and reduced levels of alanine transaminase and aspartate aminotransferase in the serum (Fig. 6*D*). Immunohistochemistry (IHC) staining of the lung sections confirmed decreased caspase-1 activation after 8A treatment (Fig. 6*E*).

**Figure 7:**
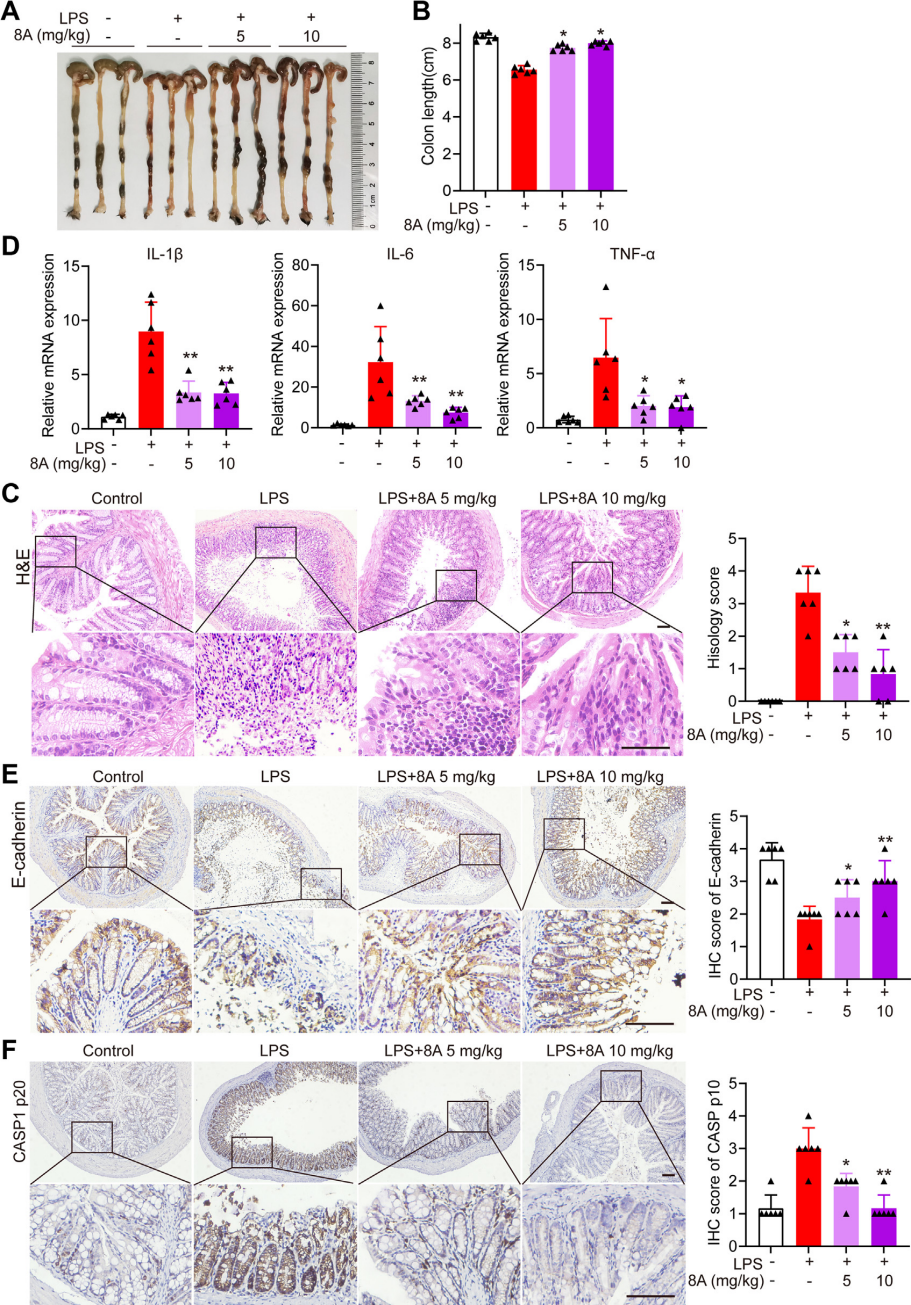
**8A decreased colon damage in mice with LPS-induced endotoxemia.** After the LPS challenge for 24 h, mice were sacrificed, following which colon damage was evaluated. *A* and *B*, by colon length; *C*, by H&E staining (*C*). *D*, levels of pro-inflammatory cytokines in colon tissue were determined using RT-PCR. *E*, paraffin-embedded colon tissue sections from each group were stained for E-cadherin. *F*, paraffin-embedded colon tissue sections from each group were stained for CASP1 p20. Data in *B* and *E* were presented as the mean ± SD of six mice. Data in *D–F* were expressed of mean ± SD of five fields per mouse in each group. ∗*p* < 0.05, ∗∗*p*< 0.01 *versus* LPS alone treated group. Scale bar 50 μm in *C*, *E* and *F*. LPS, lipopolysaccharide.

LPS treatment also caused colon damage in mice, manifested by colon shortening (Fig. 7, *A* and *B*), loss of mucosal epithelial cells (Fig. 7, *D* and *E*), and elevated expression of inflammatory cytokines (Fig. 7*E*), all of which were ameliorated following 8A administration. Moreover, 8A administration markedly suppressed CASP1 activation in the LPS-treated mice (Fig. 7*F*). The data presented in Figure 5, Figure 6, Figure 7 indicate that 8A treatment significantly alleviated LPS-induced endotoxemia in mice by inhibiting the activation of the NLRP3 inflammasome.

### 8A alleviates MSU-induced peritonitis and gouty arthritis in mice

**Figure 8:**
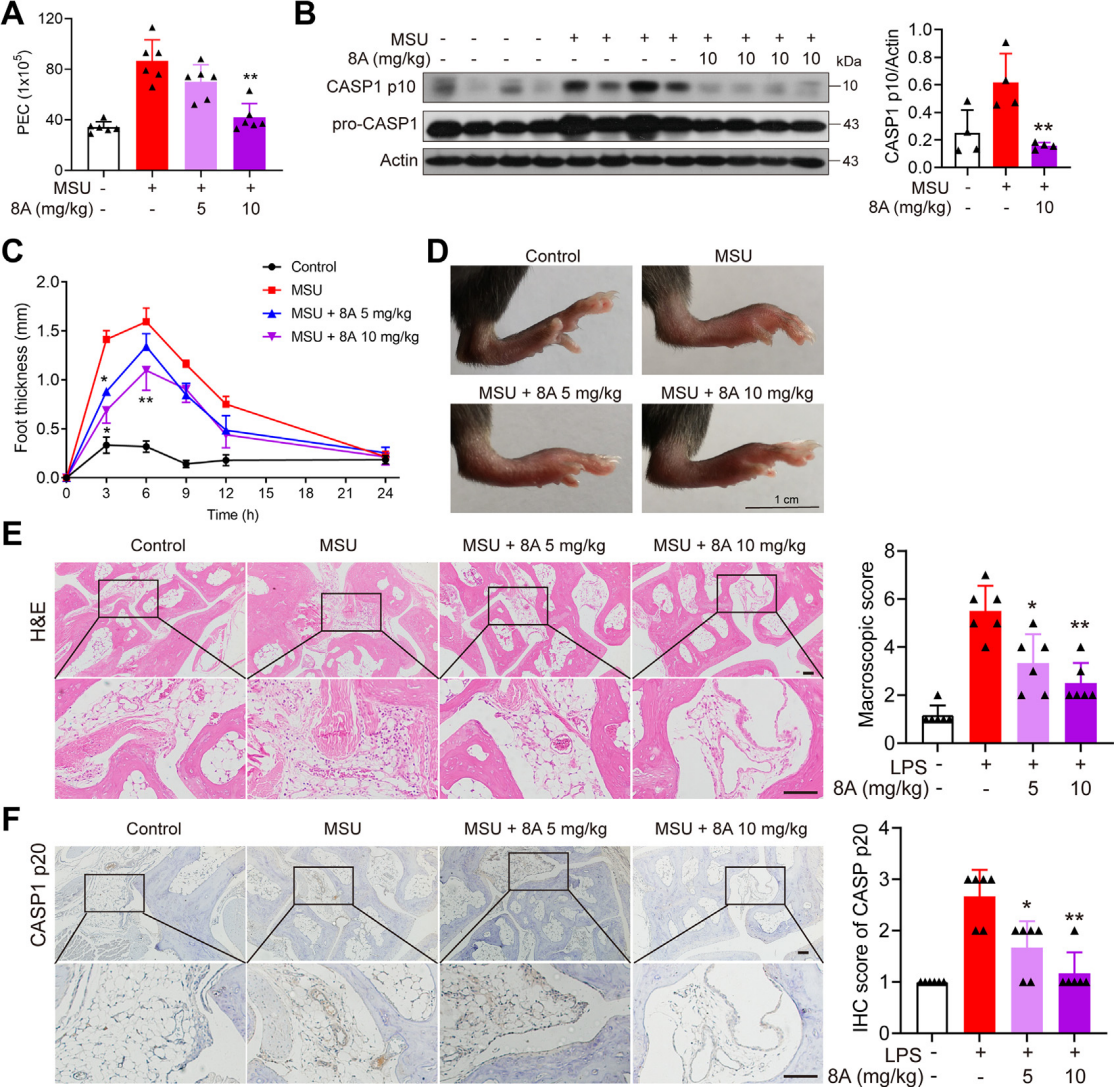
**8A alleviated MSU-induced peritonitis and arthritis.***A* and *B*, mice were i.g. with indicated doses of 8A for 3 days (once a day) before i.p. injection of MSU (1 mg MSU crystals dissolved in 0.5 ml sterile PBS). After 6 h, the mice were euthanized, and the peritoneal cavities were lavaged with 3 ml cold PBS. Peritoneal exudate cells (PECs) were counted, and the expression of CASP1 in PEC was determined by Western blotting. *C–E*, foot paw thickness changes were measured at different times after MSU injection (*C*). After MSU injection for 6 h, the manifestation of foot paws was recorded (*D*) and subjected to H&E staining (*E*). *F*, CASP1 p20 expression was analyzed by immunochemical staining. Data in *A* and *C* were presented as the mean ± SD of six mice. Data in *B* were presented as the mean ± SD of four mice. Data in *E* and *F* were expressed as the mean ± SD of five fields per mouse in each group. ∗*p* < 0.05, ∗∗*p* < 0.01 *versus* MSU group. Scale bar 100 μm in *E* and *F*. MSU, monosodium urate.

We further examined the effects of 8A on MSU-induced peritonitis and arthritis in the mice. Intraperitoneal injection of MSU crystals in mice elicited a robust inflammatory response, characterized by massive peritoneal infiltration of immune cells (Fig. 8*A*) and heightened levels of caspase-1 cleavage in peritoneal exudate cells, documented by immunoblotting (Fig. 8*B*). These immune responses were found to be significantly suppressed by 8A treatment. Similarly, ankle swelling, tissue damage, and caspase-1 activation triggered by MSU injected into the hindfoot of mice were inhibited by 8A (Fig. 8, *C*–*F*). These findings indicate that 8A treatment can reduce inflammation in mice by inhibiting NLRP3 inflammasome activation.

## Discussion

To the best of our knowledge, this is the first study to describe a small molecular weight compound that can target ASC for NLRP3 inflammasome inhibition. By targeting ASC, 8A suppressed the assembly of the NLRP3 inflammasome, thereby attenuating inflammatory diseases, including endotoxemia and gouty arthritis in mice. Moreover, the unique mechanism by which 8A inhibits multiple inflammasomes indicates its potential application in treating a broad group of inflammatory disorders.

A few small-molecular-weight compounds have been reported to indirectly or directly inhibit the activation of NLRP3. For example, our previous study found that the natural products andrographolide, asiatic acid, and the synthetic compounds Fc11a-2 and AI44 inhibited NLRP3 inflammasome activation and thus prevented colitis and LPS-induced sepsis in mice (17, 21). Furthermore, omega-3 fatty acids and beta-hydroxybutyrate have been shown to prevent inflammation and metabolic disorders *via* inhibition of NLRP3 inflammasome activation (22, 23), whereas MCC950, RRx-001, oridonin, and tranilast can directly target NLRP3 to alleviate inflammasome-driven diseases (24, 25, 26, 27, 28, 29). A novel anti-ASC monoclonal antibody, IC100, has been reported to improve the symptoms of experimental autoimmune encephalomyelitis in mice (30), and IC100 has been found to inhibit the ASC component of ASC specks, thereby disrupting their structure and function and preventing the perpetuation of the massive inflammatory response.

ASC containing a caspase recruitment domain is an essential adaptor protein for recruiting procaspase 1 into inflammasomes, including NLRP3, AIM2, and NLRP1. Genetic ablation of ASC has been shown to almost completely abolish caspase 1 activation and secretion of IL-1β and IL-18 (31, 32). It has also been reported that in the resting state, ASC localizes primarily to the nucleus of macrophages, whereas upon stimulation with certain danger-associated molecular patterns or pathogen-associated molecular patterns, ASC is rapidly translocated to the cytosol, forming specks that serve as the basis for inflammasome complex assembly and caspase-1 activation (33). In the present study, we similarly confirmed that in resting BMDMs, ASC is partly nuclear-localized and subsequently translocated to the cytosol in response to ATP or MSU stimulation. After artificially sequestering ASC within the nucleus by fusing ASC with nuclear localization sequences, inflammasome-mediated maturation of IL-1β was completely abrogated (33), indicating that preventing the redistribution of ASC into the cytosol might be an effective strategy for inactivating inflammasomes. Moreover, similarly to the aforementioned anti-ASC antibody IC100, we found that the small-molecular-weight compound 8A could bind to ASC and reduce ASC speck formation, alleviating of inflammatory diseases. Compound 8A is derived from spirodalesol, a skeletally undescribed and biologically active polyketide produced by the fungus *D. eschscholzii* (16). Compared with the IC100 antibody, 8A has several merits, including a relatively low dose (10 mg/kg) and convenience of production and oral administration.

In conclusion, the findings of this study indicate that spirodalesol analog 8A alleviates murine sepsis and gouty arthritis, with the underlying mechanism conceivably involving the targeting of ASC to induce inflammasome inactivation. Our findings thus provide evidence that ASC could serve as a reliable target in therapeutic applications for treating sepsis or other inflammasome-driven inflammatory diseases. Therefore, 8A should be considered a lead compound worthy of further development.

## Experimental procedures

### Mice

Six- to eight-week-old female C57BL/6 mice were purchased from GemPharmatech Co Ltd. Animal welfare and experimental procedures were performed in strict accordance with the Guide for the Care and Use of Laboratory Animals (National Institutes of Health) and approved by the Animal Care and Use Committee of Nanjing University. All efforts were made to minimize animals’ suffering and to reduce the number of animals used.

### Reagents

The chemical structures of spirodalesol and its analogs are listed in Fig. S1. Compounds were dissolved at a concentration of 30 mM in 100% DMSO as a stock solution (stored at −20 °C) and diluted with a medium when used. The final concentration of DMSO did not exceed 0.1% throughout the study (all control groups contained 0.1% DMSO). LPS from *Escherichia coli* (0111: B4), phorbol myristate acetate (PMA), LPS, and ATP were purchased from Sigma-Aldrich. Alanine transaminase and aspartate aminotransferase activity assay kits were purchased from the Nanjing Jiancheng Bioengineering Institute. RPMI-1640, FBS, Alexa Fluor 546 donkey anti-rabbit IgG, and Alexa Fluor 488 donkey anti-mouse IgG (H + L) were purchased from Life Technology. Anti-CD11b-PE was purchased from eBioscience. Anti-p-p65, anti-p65, anti-NLRP3, and anti-CASP1 antibodies were purchased from Cell Signaling Technology. Anti-cleaved CASP1 (p20) (cleaved-Asp210) was purchased from Tigergene. Anti-ASC was purchased from Santa Cruz. ELISA kits for murine TNF-α, IL-1β, IL-6, and human IL-1β were purchased from Dakewe Biotech Co Ltd. The FAM-FLICA CASP1 assay KIT was purchased from Immunochemistry Technologies, LLC. MCC950 (T3701) was purchased from Target Molecule Corp. All other chemicals were purchased from Sigma-Aldrich.

### Cell culture

THP1 and 293T cell lines were purchased from the Shanghai Institute of Cell Biology and maintained in RPMI 1640 medium, supplemented with 100 U/ml of penicillin, 100 μg/ml of streptomycin, and 10% fetal calf serum in a humidified 5% (v/v) CO_2_ atmosphere at 37 °C. BMDMs were harvested according to the following procedures. Bone marrow cells were isolated from C57BL/6 mice and cultured with DMEM supplemented with 10% fetal bovine serum and 20 ng/ml macrophage colony-stimulating factor (Nanjing Epotobiotech). Culture fluid was exchanged to fresh culture medium every 3 days. Under these conditions, adherent macrophages were obtained within 7 to 8 days. The cells were harvested and seeded on 24-well plates. After culturing for 6 h without macrophage colony-stimulating factor, the cells were used for the experiments as BMDM.

### MTT assay

PMA-differentiated THP1 cells were treated with 8A at the indicated concentrations. After 24 h, MTT (1 mg/ml) was added for 3 h, followed by dimethyl sulfoxide to dissolve the formazan product. The cell viability was measured using a 96-well plate reader at 570 nm.

### FAM-FLICA caspase 1 assay

PMA-differentiated THP1 cells were stimulated with 100 ng/ml LPS for 3 h, followed by 1 h incubation with 8A at the indicated concentrations and then treated with ATP or MSU. The cells were collected, stained with FLICA, and analyzed using a flow cytometer.

### LPS-induced endotoxemia in mice and treatment

For survival monitor experiment, C57BL/6 (18–20 g, female) mice were administered LPS at 10 mg/kg (i.p.) with or without 8A treatment (i.g. 5 and 10 mg/kg), and survival was monitored continuously for 60 h (n = 10 per group). For tissue collection experiment, serum and tissue samples from the lungs, liver, and colon were collected 24 h after LPS and 8A administration for analysis.

### MSU-induced peritonitis and gouty arthritis

For the peritonitis model, C57BL/6 (18–20 g, female) were treated with the indicated doses of 8A (5 and 10 mg/kg) for 3 days (orally administered, once a day) before intraperitoneal injection of MSU (1 mg MSU crystals dissolved in 0.5 ml sterile PBS). After 2 h, the mice were killed, and the peritoneal cavities were lavaged with 3 ml cold PBS. The number of peritoneal exudate cells was counted using a Countstar BioMed machine (ALIT Life Sciences), and caspase-1 was examined by Western blot. For the gouty arthritis model, C57BL/6 mice (18–20 g, male) were orally administered with 8A (5, 10 mg/kg) 3 days (once a day). One hour after the last administration, MSU (0.5 mg MSU crystals dissolved in 20 μl sterile PBS) was administrated by intra-articular injection. An electronic caliper was used to measure joint sizes at the indicated time point.

### Cytokine analysis by ELISA

The levels of IL-1β, IL-6, and TNF-α in the serum and cell culture supernatant were quantified using ELISA kits (Dakewe).

### IF and IHC

IF of BMDM and IHC on paraffin-embedded tissue sections were performed as reported (34, 35). Briefly, cells or tissue sections were fixed in 4% formaldehyde, permeabilized in 0.5% Triton-100 in PBS or saponin (Beyotime), and incubated in a blocking solution containing 5% BSA. For IF, samples were stained with primary antibodies at 4 °C overnight, followed by incubation with fluorescently labeled secondary antibodies. Coverslips were mounted in an antifade solution containing DAPI and imaged using a confocal laser scanning microscope or fluorescence microscope (Leica TCS SP8-MP). For IHC, sections were incubated with specific primary antibodies for 2 h at room temperature, then incubated with streptavidin-HRP for 40 min, stained using DAB substrate, and counter-stained with hematoxylin. Images were acquired using microscope (Olympus). Histological evaluation of H&E-stained colonic sections was graded in a blinded manner as follows (36): 0, no signs of inflammation; 1, low leukocyte infiltration; 2, moderate leukocyte infiltration; 3, high leukocyte infiltration, moderate fibrosis, high vascular density, thickening of the colon wall, moderate goblet cell loss, and focal loss of crypts; and 4, transmural infiltrations, massive loss of goblet cells, extensive fibrosis, and diffuse loss of crypts. The quantification of IHC was performed using IHC Profiler (https://sourceforge.net/projects/ihcprofiler) and scored at four levels (4, high positive; 3, positive; 2, low positive; and 1, negative). The acquired data are expressed as a histogram of each group’s mean ± SD of five fields per mouse.

### Western blotting

Western blotting was performed as previously reported (21). Briefly, proteins were extracted using lysis buffer, separated by SDS–polyacrylamide gel electrophoresis (PAGE), and electrophoretically transferred onto polyvinylidene difluoride membranes. The membranes were probed with antibodies overnight at 4 °C and then incubated with a horseradish peroxidase–coupled secondary antibody. Detection was performed using the Lumi-GLO chemiluminescent substrate system.

### Cellular thermal shift assay

THP1 cells were incubated with DMSO or 8A (3 μM) for 2 h, collected, and subjected to a CETSA (37). Briefly, incubated cells were equally divided into 10 parts, and each part was heated for 3 min at different temperature (43, 46, 49, 52, 55, 58, 61, 64, 67 and 70 °C). The heated cells were kept at −80 °C for 12 h, transferred to room temperature for 5 min, and this sequence was repeated one more time. Cell lysates were extracted by centrifugation at 20,000*g* for 20 min. ASC expression was detected by Western blotting. For the *in vivo* thermal shift assay, C57BL/6 mice were administered 10 mg/kg 8A (once a day for 3 days). Peritoneal macrophages were collected and subjected to CETSA.

### Microscale thermophoresis

MST assays were performed using a Monolith NT.115 (Nano Temper). The cell lysates containing ASC-EGFP was used in this study. Proteins were extracted from the cells transfected with the ASC-EGFP plasmid. Fluorescence intensity was detected using Pico Red channel or Nano Blue channel, and the protein was diluted to an appropriate concentration for subsequent experiments. The diluted compounds (16 concentration gradients, 1:1 dilution) were respectively mixed with the protein and detected by capillary aspiration. MO. Affinity analysis software was used for the combined analysis.

### ASC oligomerization assay

ASC oligomerization was detected as described previously (34). BMDMs were washed with ice-cold PBS and lysed with NP-40 at 4 °C for 30 min. The lysates were centrifuged at 330*g* for 10 min at 4 °C to obtain pellets. After being washed with 1 ml of ice-cold PBS, the pellets were resuspended in 500 μl of ice-cold PBS. Disuccinimidyl suberate (2 mM) was added to the resuspension solution, which was then incubated at room temperature with rotation for 30 min. The mixture was centrifuged at 4 °C and 330*g* for 10 min. Sample buffer (30 μl) was used to lyse the crosslinked pellets, and immunoblotting was performed.

### NLRP3 oligomerization assay

NLRP3 oligomerization assay was performed as previously reported (38). After the indicated treatment, cells were suspended in Hepes buffer containing 5 mM EDTA and protease inhibitors. Cells were lysed by passage (20 times) using a 21-gauge needle. The nuclei and unlysed cells were removed by centrifugation at 900*g* for 8 min. The supernatants were collected and centrifuged at 6200*g* for 8 min. The pellet fractions were suspended in 200 μl of Hepes buffer and then cross-linked for 1 h at 37 °C with 2 mM fresh suberic acid bis (3-sulfo-N-hydroxysuccinimide ester) sodium salt (BS3; Sigma-Aldrich, 82,436–77–9). The cross-linked pellets were dissolved in the SDS loading buffer, and immunoblotting was performed to detect NLRP3 oligomerization.

### Statistical analysis

The results are expressed as the mean ± SD of three independent experiments, and each experiment included triplicate sets. Data were statistically evaluated by one-way ANOVA followed using Dunnett’s test between the control group and multiple-dose groups. The level of significance was set at a *p* value of 0.05.

## Data availability

All data are contained within the manuscript.

## Supporting information

This article contains supporting information.

## Conflict of interest

The authors declare no conflict of interests with the contents of the article.
