# Supplementary material for: Spirodalesol analog 8A inhibits NLRP3 inflammasome activation and attenuates inflammatory disease by directly targeting adaptor protein ASC
Source: J Biol Chem. 2022 Nov 12;298(12):102696. doi: 10.1016/j.jbc.2022.102696 (PMC9730227; doi:10.1016/j.jbc.2022.102696)
Supplement: Supplemental Figures S1–S5 [file mmc1.docx]

**
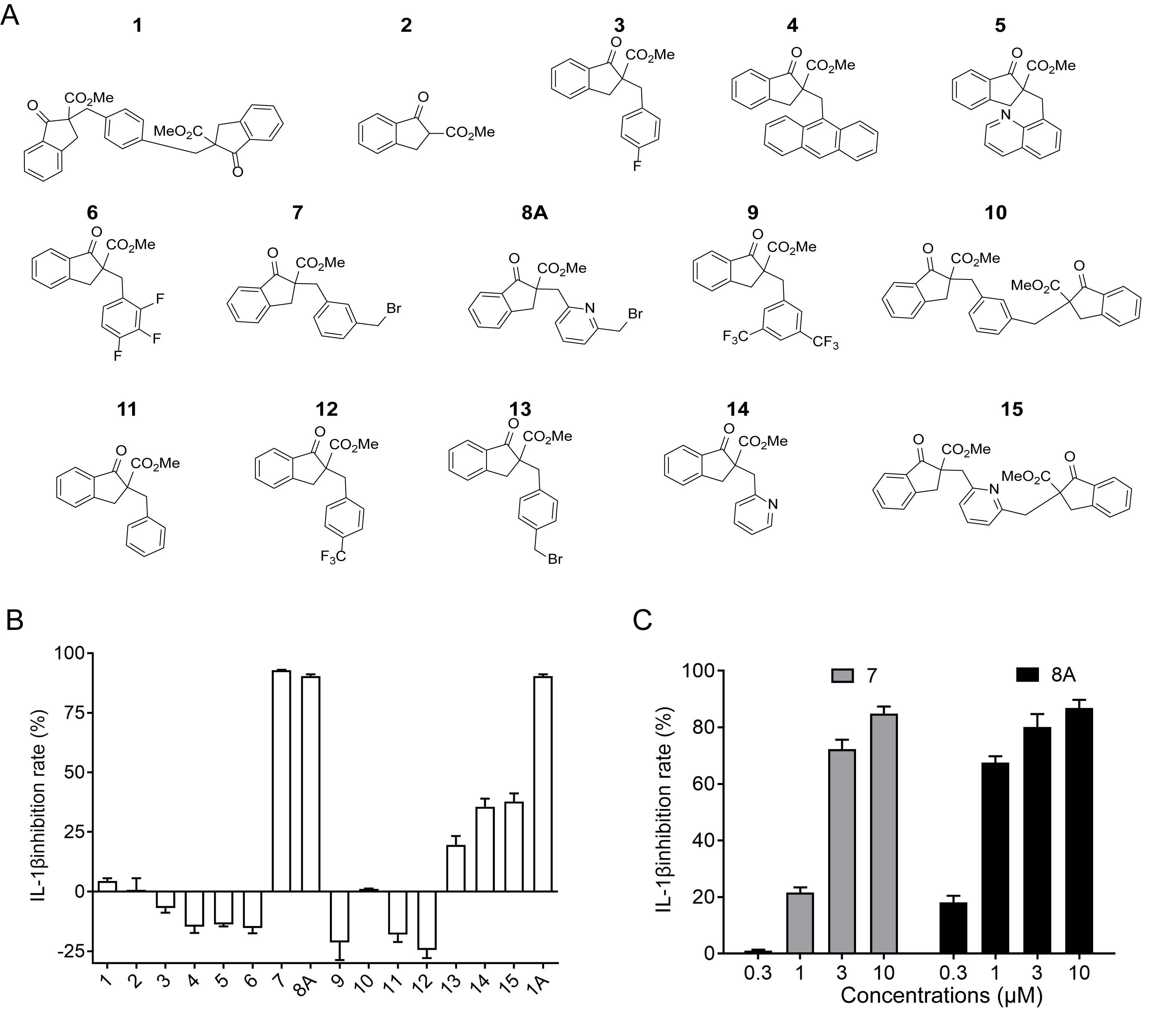
**

**Supplementary fig. s1. The effects of small compounds on IL-1β production in THP-1-derived macrophages activated by LPS plus ATP**. (A) Structural formula of a serial of AI compounds. (B) LPS-primed THP-1 cells were treated with a series of compounds at a dose of 10 μM for 1 h, respectively, following by 5 mM ATP treatment for 1 h. IL-1β in the supernatant was analyzed by ELISA. (C) LPS-primed THP-1 cells were treated with various concentrations of compound 7 and 8A for 1 h, respectively, following by 5 mM ATP treatment for 1 h. IL-1β in the supernatant was analyzed by ELISA. Data are presented as mean ±SEM of three independent experiments. **P*<0.05, ***P*<0.01 vs. LPS plus ATP group.


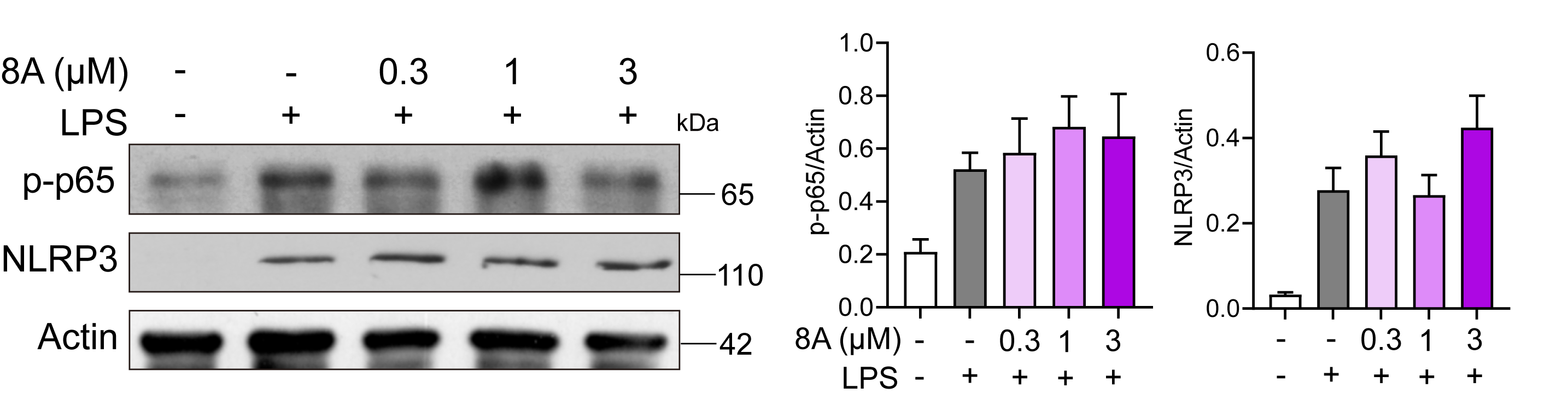


**Supplementary fig. s2. 8A did not alter NF-κB activation induced by LPS.** BMDM were treated with 100 ng/ml LPS with or without 8A for 1 h. Expression of p-p65 and NLRP3 were determined by western blot.


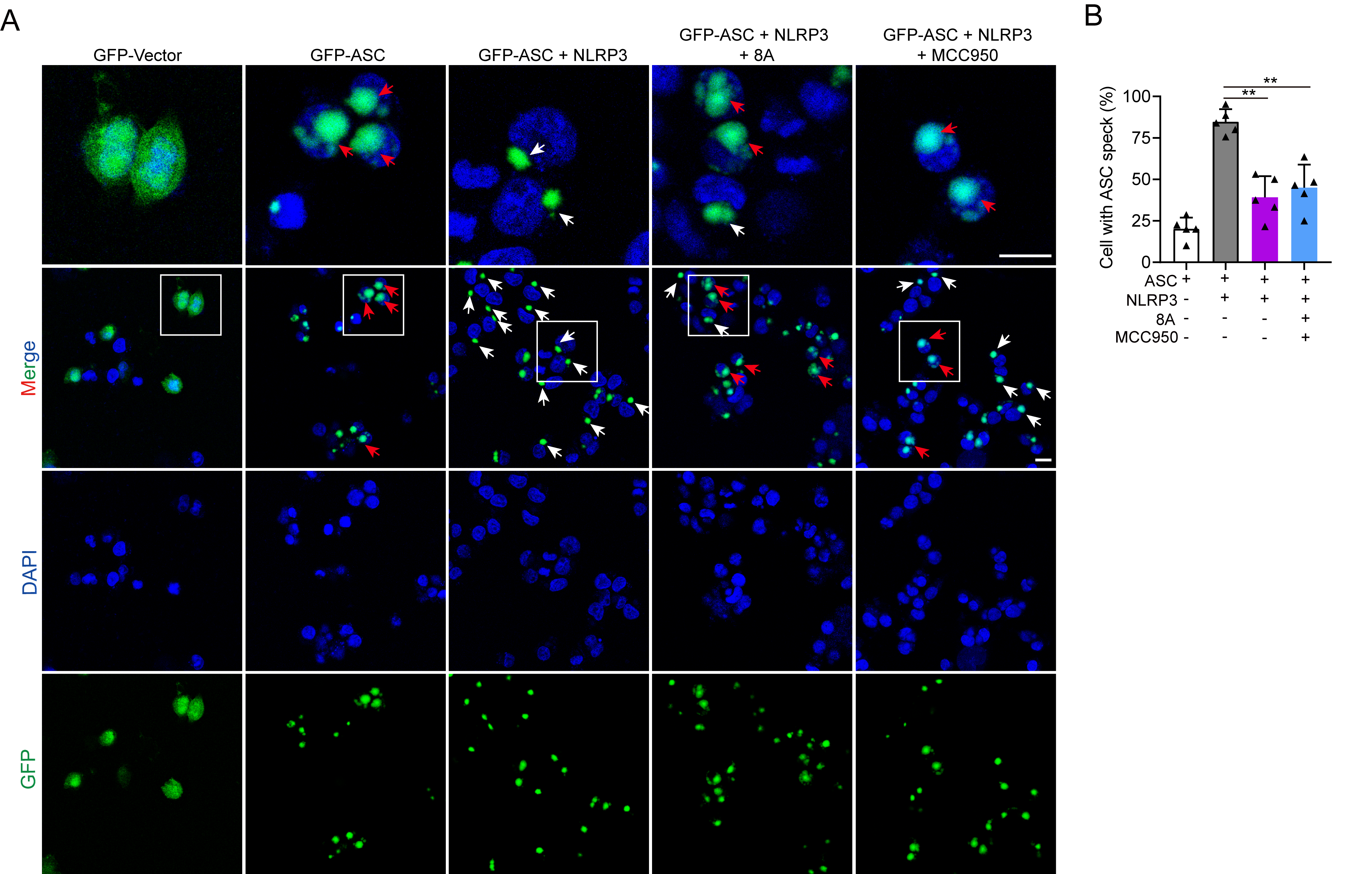


**Supplementary fig. s3.** **8A inhibited ASC speck formation.** (A, B) ASC-GFP-encoding plasmid was transfected into 293T cells with or without NLRP3-encoding plasmid. Twenty hours after transfection, the cells were treated with 8A (3 μM) or MCC950 (0.3 μM) for another 3 h. ASC speck formation (indicated by white arrow) or ASC nucleic location (indicated by red arrow) was determined by immunofluorescence. Cells with ASC speck were counted in five fields from every group and expressed as the mean ± SD. * *P* <0.05, ** *P* <0.01 vs. as indicated. Scale bar 10 μm.

**
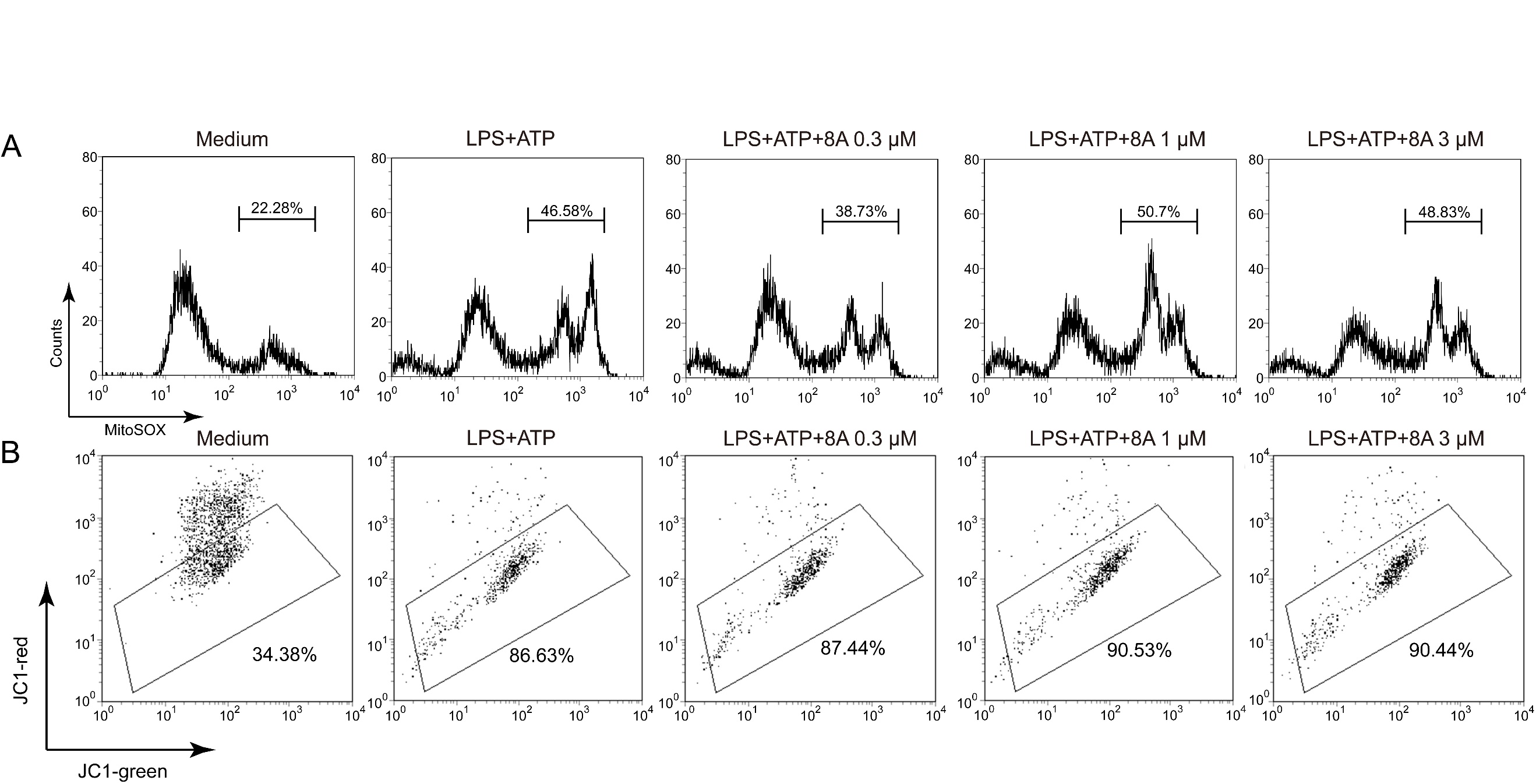
**

**Supplementary fig. s4. Effects of 8A on ROS** **production and mitochondria membrane potential in THP-1-derived macrophages.** THP-1 cells, pretreated with 500 nM PMA for 3 h, were cultured with 100 ng/ml LPs for 3 h (below referred to as LPs-primed ThP-1 cells), then cells were treated with 8A (0.3, 1, or 3 μM) for 1 h, followed by 1 h incubation with 5 mM ATP. The ROS level and mitochondrial membrane potential were determined by DCFH-DA (A) and JC-1 staining (B).


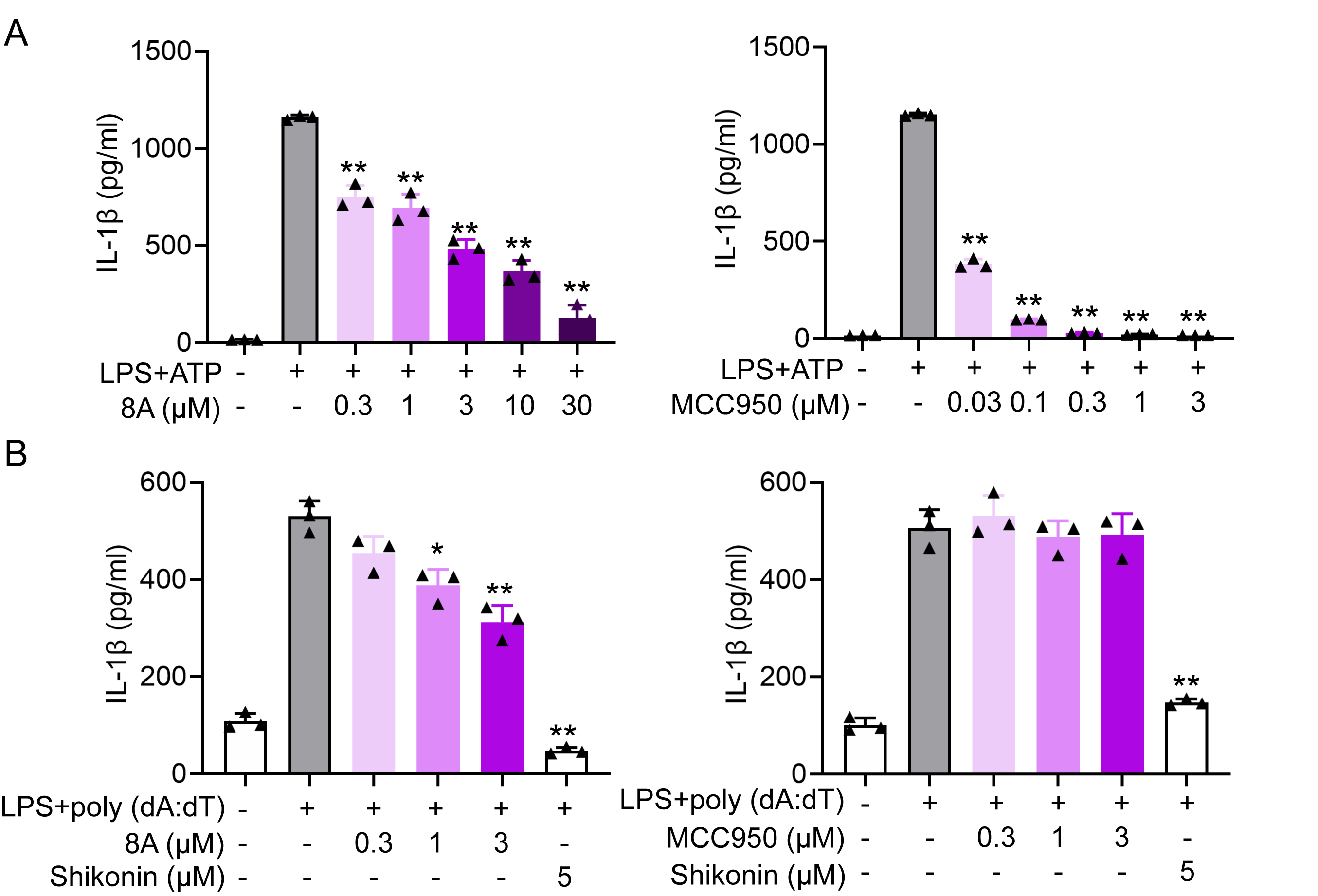


**Supplementary fig. s5. Effects of 8A on AIM2 inflammasome activation.** LPS primed BMDM cells were treated with indicated doses of 8A, MCC950 or shikonin for 1 h, followed by ATP (5 mM) stimulation for 1 h (A), poly(dA:dT) (0.5 μg/ml) for 2 h (B). IL-1β in supernatant were determined by ELISA. Data are presented as the mean ± SD of three independent experiments. * *P* < 0.05, ** *P* < 0.01 vs. LPS + ATP or LPS + poly(dA: dT).
